# Modulation of Acupuncture on Cell Apoptosis and Autophagy

**DOI:** 10.1155/2017/8268736

**Published:** 2017-11-27

**Authors:** Dan Luo, Rui Chen, Feng-xia Liang

**Affiliations:** ^1^Department of Traditional Chinese Medicine, Union Hospital, Tongji Medical College, Huazhong University of Science and Technology, No. 1277 Jiefang Street, Wuhan, Hubei 430022, China; ^2^Department of Acupuncture and Moxibustion, Hubei University of Traditional Chinese Medicine, No. 1 Tanhualin Street, Wuhan, Hubei 430060, China

## Abstract

Acupuncture has been historically practiced to treat medical disorders by mechanically stimulating specific acupoints. Despite its well-documented efficacy, its biological basis largely remains elusive. Recent studies suggested that cell apoptosis and autophagy might play key roles in acupuncture therapy. Therefore, we searched PubMed, Embase, Web of Science, and China National Knowledge Infrastructure (CNKI), aiming to find the potential relationship between acupuncture and cell apoptosis and autophagy. To provide readers with objective evidence, some problems regarding the design method, acupoints selection, acupuncture intervention measure, and related diseases existing in 40 related researches were shown in this review. These findings demonstrated that acupuncture has a potential role in modulating cell apoptosis and autophagy in animal models, suggesting it as a candidate mechanism in acupuncture therapy to maintain physiologic homeostasis.

## 1. Introduction

Acupuncture is a key component of traditional Chinese medicine (TCM) and a main form of alternative medicine [1]. The therapy functions by means of stimulating certain acupoints in the human body to activate meridians and collaterals and regulate the function of Zang-Fu organs and Qi and blood [2]. Acupuncture, with many categories such as manual acupuncture (MA), electroacupuncture (EA), laser acupuncture, and acupoint injection, has turned out to be relatively safe with few adverse effects [3].

Apoptosis and autophagy are two important cellular processes which control cell survival or death [4] and are also considered as a balanced response to pathogens and other immune stimuli that play an important role in maintaining physiologic homeostasis [5]. Apoptosis- and autophagy-related mechanisms have been increasingly valued in neurological diseases [6], diabetes mellitus [7], and cancer [8]. However, there are few effective and safe ways to regulate cell apoptosis and autophagy in clinical practice right now.

The treatment of acupuncture in diseases like nerve injury has been extensively studied for a long time [9]. Acupuncture could regulate multiple molecules and signaling pathways that lead to excitoxicity, oxidative stress, inflammation, and neurons death and survival and also promote neurogenesis, angiogenesis, and neuroplasticity after ischemic damage [10].

Based on recent studies, the mechanism of acupuncture to treat medical disorders has a high degree of overlap with cell apoptosis and autophagy, which may provide a new direction for the clinical application and basic research. Up to now, there has been no review to clarify the potential relationship between acupuncture and cell apoptosis and autophagy. Herein, we performed a review, in particular focused on the therapy of acupuncture, including design method, acupoints selection, acupuncture intervention measure, and related diseases, trying to find out the detailed mechanism and objective evidence for modulation of acupuncture on cell apoptosis and autophagy.

## 2. Materials and Methods

Relevant studies were identified from the online electronic databases PubMed, Embase, Web of Science, and China National Knowledge Infrastructure (CNKI). Search terms consisted of three groups: apoptosis and autophagy* (an important mechanism for the treatment of diseases or maintaining homeostasis)*, intervention (acupuncture and other related terms), and study type* (randomized controlled trial and other related terms)*. Methodological quality of individual studies was assessed by (1) risk of bias, (2) inconsistency, (3) indirectness, (4) imprecision, and (5) publication bias. Finally, 40 articles were identified in this review, which were published in English only and provided full text until May 2017.

## 3. Results

**Figure 1:**
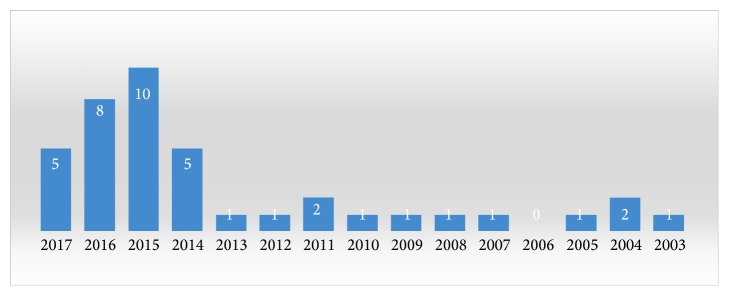
The number of articles published every year from 2003 to 2017.

All 40 articles were randomized controlled trials for animal studies. There was a huge change regarding the literature published from 2003 to 2017. During the first decade, only a few reports were published each year, with a substantial increase in the last four years (Figure 1).

Acupoints selection was performed according to both clinical reports and traditional Chinese medicine theory. Most articles were related to neurological diseases like ischemia-reperfusion (I/R) that caused some certain acupoints to be used more frequently than others such as GV20* (Baihui)* and ST36* (Zusanli)*.

All results indicated that acupuncture has the effect of suppressing cell death* (TUNEL assay or other tests, p < 0.05 or 0.01)*, inhibiting inflammation, or removing pathologic products though regulating cell apoptosis and autophagy* (p < 0.05 or 0.01)*. The mechanism of acupuncture in modulation of cell apoptosis and autophagy, which was associated with regulating the expression of Bcl-2/Bax, caspase family, Fas/FasL, c-Fos, TNF-*α*, or NF*κ*B, has been extensively and intensively studied from biological and immunological perspectives. Detailed information of all researches is shown in Table 1.

## 4. Discussion

To our knowledge, this is the first review to explore the efficacy of acupuncture for modulating cell apoptosis and autophagy. Based on the biological and immunological results in 40 studies, it is indicated that acupuncture could regulate the expression of Bcl-2/Bax, caspase family, Fas/FasL, c-Fos, TNF-*α*, and NF*κ*B, which modulated cell apoptosis and autophagy to reduce cell death* (TUNEL assay or other tests, p < 0.05 or 0.01)* in different pathological states especially ischemic stroke. Most studies suggested that acupuncture suppresses cell apoptosis. However, it is interesting that acupuncture plays a dual role in regulating autophagy. Acupuncture could not only promote autophagy to remove pathology products, but also inhibit autophagy against cell death in different periods of diseases. All the results suggested that acupuncture on cell apoptosis and autophagy does not have specificity and involves numerous pathways.

Acupuncture has been known as an effective therapy in neurobiology [11] and immunology [12], but the mechanism is still unclear. It is recognized that cell apoptosis and autophagy are associated with more and more diseases. Apoptosis, a key regulator of tissue homeostasis, is tightly regulated with the interaction of activating and inhibitory pathways. Aberrant induction of cell apoptosis may result in neurodegenerative, chronic, inflammatory, and autoimmune diseases, among others [12]. Autophagy, an intracellular process in which cytoplasmic materials are transported by double-membraned autophagosomes to lysosomes for degradation [13], is a highly conservative biological degradation pathway that plays essential roles in cell homeostasis, development, and survival [14]. So, we hypothesize that cell apoptosis and autophagy might play key roles in acupuncture treatment of diseases.

The latest researches reported that epigenetic modification plays a great role in cell apoptosis and autophagy [15, 16]. SIRT1* (silent mating type information regulation 2 homolog 1)* is an NAD-dependent deacetylase which has a deacetylation activity in the modulation of cell stress signals via epigenetics [17], and the deacetylation of histone via SIRT1 was considered as an important intervention for apoptosis and autophagy [18]. Our previous study confirmed that acupuncture induces the activation of SIRT1 [19], so it is expected that acupuncture-SIRT1-epigenetic modification to modulate cell apoptosis and autophagy will be investigated in the near future.

There are some limitations to this review. Firstly, our search only included English articles and excluded those articles published in other languages. Although we have performed comprehensive literature search, the total number of studies and the total sample size were too small to be reliable. Secondly, articles which reported negative results may not be popular to publish so that the effectiveness of published articles would be better than those unpublished, which may cause publication bias. Thirdly, due to the lack of repeat test under the same conditions, the conviction of the conclusion is still insufficient. Based on the above limitations, detailed results of each study were shown in this review to provide objective evidence on acupuncture modulation of apoptosis and autophagy.

## 5. Conclusion

In conclusion, studies suggested that acupuncture has a potential role in modulating cell apoptosis and autophagy in animal models, suggesting it as a candidate mechanism in acupuncture therapy to maintain physiologic homeostasis. However, detailed mechanisms were still not very legible and the publication bias may reduce persuasiveness of positive results. Hence, more high-quality randomized controlled trials to clarify the role of the relevant mechanisms are needed in the future.

## Figures and Tables

**Table 1:** Modulation of acupuncture on cell apoptosis and autophagy.

Diseases related	Models	Control group	Acupoints	Effects of acupuncture intervention	Study
Alzheimer's disease (AD)	Intrahippocampally injected A*β*_1–40_ model, rats	Non-EA	Du20, BL23, EA	(1) The Hoechst 33342 positive apoptotic cells decreased (*p* < 0.01) (2) The protein expression of Bcl-2 was increased (*p* < 0.01) (3) The protein expression of Bax was decreased (*p* < 0.05) (4) The mRNA expression of Notch1 and hes1 was decreased (*p* < 0.05 and *p* < 0.01)	[20]

Brain function disorder	Normal, rats	Non-MA	SJ5, MA	Both the mRNA and protein expression of Bcl-2 were increased (*p* < 0.01)	[21]

Central poststroke pain (CPSP)	The CPSP model, single collagenase injection into the left ventral posterolateral nucleus of the thalamus, rats	Non-EA	GV20, ST36, EA	(1) The TUNEL-positive cells decreased (*p* < 0.05) (2) The expression of COX-2, *β*-catenin, and NK-1R was decreased (*p* < 0.01)	[22]

Cervical spondylosis (CS)	Inducing cervical IVD degradation through unbalanced dynamic and static forces, rats	Non-EA	GV14, EA	(1) The TUNEL-positive cells decreased (*p* < 0.05) (2) The expression of TNF-*α*, TNFR1, and caspase-8 was decreased (*p* < 0.01) (3) Both the mRNA and protein expression of integrin-*β*1 and Akt were increased (*p* < 0.05)	[23]

Cerebral palsy (CP)	Hypoxia-ischemia (HI), rats	Non-MA	GV20, Ex-HN1, MA	(1) The TUNEL-positive cells decreased (*p* < 0.01) (2) The protein expression of Bcl-2 was increased (*p* < 0.05) (3) The protein expression of Bax, caspase-3, and caspase-9 was decreased (*p* < 0.05)	[24]

Depressive-like and anxiety-like behaviors	The CUS model, rats	Non-EA	DU20, GB34, EA	(1) The BrdU-positive cells (ANPs) obviously increased (*p* < 0.05) (2) The Hoechst 33342 positive apoptotic cells (QNPS) decreased (*p* < 0.05)	[25]

Heroin addiction	Heroin readdiction was produced through repeated exposure and detoxification, rats	Non-EA	GV20, GV14, EA	(1) The protein expression of Bcl-2 was increased (*p* < 0.01) (2) The protein expression of Bax was decreased (*p* < 0.01)	[26]

Ischemic stroke	Middle cerebral artery occlusion (MCAO) model, rats	Non-EA	LU5, LI4, ST36, SP6, EA	(1) The TUNEL-positive cells decreased (*p* < 0.01) (2) The protein expression levels of p-ERK were increased (*p* < 0.05)	[27]

Ischemic stroke	Middle cerebral artery occlusion (MCAO) model, rats	Non-EA	GV20, EA	(1) Both the mRNA and protein expression of NDRG2 were inhibited (*p* < 0.01) (2) The TUNEL-positive cells decreased (*p* < 0.05)	[28]

Ischemic stroke	Transient global ischemia, gerbils	Non-MA	ST36, LI4, MA	(1) The TUNEL-positive cells obviously decreased (*p* < 0.01) (2) The BrdU-positive cells obviously increased (*p* < 0.01)	[29]

Ischemic stroke	The common carotid arteries (CCAs) were occluded using aneurysm clips for 5 min, gerbils	Non-MA	ST36, LI4, MA	(1) The TUNEL-positive cells decreased (*p* < 0.05) (2) The Fos-positive cells decreased (*p* < 0.05) (3) The caspase-3-positive cells decreased (*p* < 0.05)	[30]

Ischemic stroke	Middle cerebral artery occlusion (MCAO) model, rats	Non-EA	GV20, EA	(1) The expression of *ε*PKC was increased (*p* < 0.05) (2) The TUNEL-positive cells obviously decreased (*p* < 0.01) (3) The protein expression of Bcl-2 was increased (*p* < 0.01) (4) The protein expression of Bax was decreased (*p* < 0.01)	[31]

Ischemic stroke	Middle cerebral artery occlusion (MCAO) model, rats	Non-EA	GV20, LI4, LR3, EA	(1) The TUNEL-positive cells decreased (*p* < 0.05) (2) The protein expression of Bcl-2 was increased (*p* < 0.05) (3) The protein expression of Bax was decreased (*p* < 0.05) (4) The BrdU+/Nestin+ cells obviously increased (*p* < 0.05) (5) Both the mRNA and protein expression of MMP-9 were decreased (*p* < 0.05) (6) Both the mRNA and protein expression of TIMP-1 were increased (*p* < 0.05)	[32]

Ischemic stroke	Middle cerebral artery occlusion (MCAO) model, rats	Non-EA	GV26, EA	(1) The protein expression of LC3 and Beclin-1 was decreased (*p* < 0.05) (2) The TUNEL-positive cells decreased (*p* < 0.01) (3) The Bcl-2-positive cells decreased (*p* < 0.05)	[33]

Ischemic stroke	Middle cerebral artery occlusion (MCAO) model, rats	Non-EA	GV20, GV16, EA	(1) The ratio of cytosolic p-p38 MAPK/p38 MAPK expression was increased (*p* < 0.05) (2) The protein expression of Bcl-2 was increased (*p* < 0.05) (3) The protein expression of Bax was decreased (*p* < 0.05) (4) The expression of caspase-3 was decreased (*p* < 0.05)	[34]

Ischemic stroke	Middle cerebral artery occlusion (MCAO) model, rats	Non-EA	LI11, ST36, EA	(1) The level of LC3BII/LC3BI was decreased (*p* < 0.05) (2) The expression of mTORC1 was increased (*p* < 0.01) (3) The expression of Beclin-1 was decreased (*p* < 0.01)	[35]

Ischemic stroke	Middle cerebral artery occlusion (MCAO) model, rats	Non-EA	DU20, DU24, EA	(1) The TUNEL-positive cells decreased (*p* < 0.05) (2) The expression of NF-*κ*B p65 was decreased (*p* < 0.05) (3) The mRNA expression of Bax and Fas was decreased (*p* < 0.05)	[36]

Ischemic stroke	Middle cerebral artery occlusion (MCAO) model, rats	Non-EA	LI11, ST36, EA	(1) The TUNEL-positive cells decreased (*p* < 0.01) (2) The expression of PI3K and p-Akt was increased (*p* < 0.05) (3) Both the mRNA and protein expression of Bcl-2 were increased (*p* < 0.05)	[37]

Ischemic stroke	Model of cerebral ischemia-reperfusion, rats	Non-EA	BL3, BL17, GV20, EA	The TUNEL-positive cells decreased (*p* < 0.01)	[38]

Ischemic stroke	Global cerebral ischemia, mice	Non-EA	GV20, EA	(1) The expression of GluR2 was increased (*p* < 0.05) (2) The protein expression of Bcl-2 was increased (*p* < 0.05) (3) The protein expression of Bax was decreased (*p* < 0.05)	[39]

Ischemic stroke	Middle cerebral artery occlusion (MCAO) model, rats	Non-EA	GV20, EA	(1) The number of autophagosomes was decreased (12 h after I/S) (*p* < 0.05) (2) The TUNEL-positive cells decreased (*p* < 0.05)	[40]

Ischemic stroke	Middle cerebral artery occlusion (MCAO) model, rats	Non-EA	GV20, EA	(1) The TUNEL-positive cells decreased (*p* < 0.05) (2) The number of autophagosomes was increased (2 h after I/S) (*p* < 0.05) (3) The ratio of p-mTOR/mTOR was increased (*p* < 0.01)	[41]

Ischemic stroke	Cerebral ischemia-reperfusion model, rats	Non-EA	GV4, GV6, GV14, GV20, GV24, GV26, EA	The TUNEL-positive cells decreased (*p* < 0.01)	[42]

Intracerebral hemorrhage (ICH)	Intracranial hemorrhage model, rats	Non-MA	GV14, GV16, MA	(1) The protein expression of Bcl-2 was increased (*p* < 0.05) (2) The protein expression of Bax was decreased (*p* < 0.05) (3) The protein expression of caspase-3 was decreased (*p* < 0.05) (4) The TUNEL-positive cells decreased (*p* < 0.001)	[43]

Ischemic preconditioning (IPC)	Chest incision and 20 minutes of ischemia followed by 40 minutes of reperfusion, rats	Non-EA	PC6, CV7, EA	(1) The TUNEL-positive cells decreased (*p* < 0.05) (2) The expression of c-Fos mRNA was increased (*p* < 0.05)	[44]

Intracerebral hemorrhage	Model of intracerebral hemorrhage, rats	Non-MA	ST36, MA	(1) The TUNEL-positive cells decreased (*p* < 0.05) (2) The caspase-3-positive cells decreased (*p* < 0.05)	[45]

Intervertebral disc (IVD) degeneration	Inducing cervical IVD degradation, rats	Non-EA	DU14, LI10, EA	(1) The TUNEL-positive cells decreased (*p* < 0.01) (2) The expression of Bcl-2 was increased (*p* < 0.05) (3) The expression of Bax was decreased (*p* < 0.01) (4) The expression of caspase-3 and caspase-9 was decreased (*p* < 0.01) (5) Both the mRNA and protein expression of CrK and ERK2 were increased (*p* < 0.05)	[46]

Intervertebral disc degeneration (IVDD)	Using a custom-made external compression device to stimulate disc degeneration, rats	Non-EA	Ex-B2, EA	(1) The TUNEL-positive cells decreased (*p* < 0.05) (2) The protein expression of Bcl-2 was increased (*p* < 0.05)	[47]

Peripheral nerve injury	Model was established by mechanical clamping of the sciatic nerve stem, rats	Non-EA	GB30, EA	(1) The TUNEL-positive cells decreased (*p* < 0.05) (2) The expression of Bcl-2 was increased (*p* < 0.05) (3) The expression of Bax was decreased (*p* < 0.05)	[48]

Parkinson's disease (PD)	PD model, MPTP, 30 mg/kg/d, 5 days, mice	Non-MA	GB34, MA	(1) The expression level of LC3II was decreased (*p* < 0.05) (2) The expression level of LAMP1 was increased (*p* < 0.05)	[49]

Spinal cord ischemia-reperfusion (I/R) injury	The spinal cord I/R model received aortic arch exposure or cross-clamping for 14 min, rats	Non-EA	GV6, GV9, EX-B2, EA	(1) The protein expression of LC3 and Beclin-1 was increased (*p* < 0.05) (2) The TUNEL-positive cells decreased (*p* < 0.01) (3) The protein expression of TNF-*α*, IL-1*β*, and MMP-9 was decreased (*p* < 0.01)	[50]

Spinal cord injury (SCI)	Models were deeply anesthetized with an intraperitoneal injection of 1% pentobarbital sodium (35 mg/kg), rats	Non-EA	GV6, GV9, EA	(1) The expression of miR-214 was increased (*p* < 0.01) (2) The protein expression of Bcl-2 was increased (*p* < 0.01) (3) The protein expression of Bax was decreased (*p* < 0.01) (4) The protein expression of caspase-3 was decreased (*p* < 0.01) (5) The TUNEL-positive cells obviously decreased (*p* < 0.01)	[51]

Spinal cord injury (SCI)	Model of bladder dysfunction after SCI, rabbits	Non-EA	BL54, ST28, CV6, CV3, EA	(1) The TUNEL-positive cells obviously decreased (*p* < 0.001) (2) The expression of p-Akt and p-ERK1/2 was increased (*p* < 0.01) (3) The expression of Cyt *c* and caspase-3 was decreased (*p* < 0.01)	[52]

Spinal cord injury (SCI)	T10 segment spinal cord injury (SCI) model, rats	Non-EA	DU9, EA	(1) The expression of miR-449a was decreased (*p* < 0.01) (2) The ratio of Bax/Bcl-2 expression was decreased (*p* < 0.01) (3) The protein expression of caspase-3, TNF-*α*, and IL-1*β* was decreased (*p* < 0.01)	[53]

Sepsis (for brain injury)	Exposing cecum with ligation and puncture, rats	Non-EA	GV20, ST36, EA	(1) The TUNEL-positive cells decreased (*p* < 0.01) (2) The expression of IL-6, TNF-*α*, NF-*κ*B, and TLR-4 was decreased (*p* < 0.05)	[54]

Surgical trauma	Model of surgical trauma, rats	Non-EA	ST36, EXTRA37, EA	(1) The number of splenocytes was increased (*p* < 0.01) (2) The TUNEL-positive cells decreased (*p* < 0.05) (3) The protein expression of Fas was decreased	[55]

Surgical trauma	Model of surgical trauma, rats	Non-EA	ST36, EXTRA37, EA	(1) The apoptotic rate of the splenic lymphocytes was decreased (*p* < 0.05) (2) The protein expression of TNF-*α* and TNFR1 was decreased (*p* < 0.05) (3) The protein expression of caspase-3 and caspase-8 was decreased (*p* < 0.05) (4) The activity of JNK and NF-*κ*B was decreased (*p* < 0.05)	[56]

Transient brain ischemia	High-sustained positive acceleration (+Gz) exposure, rats	Non-EA	GV20, EA	(1) The TUNEL-positive cells decreased (*p* < 0.05) (2) The expression of caspase-3 was decreased (*p* < 0.05)	[57]

Ulcerative colitis (UC)	Model of ulcerative colitis was established by immunological methods and local stimulation, rats	Non-EA	RN6, ST25, EA	(1) The TUNEL-positive cells obviously decreased (*p* < 0.01) (2) The expression of Bcl-2 was increased (*p* < 0.01) (3) The expression of Bax was decreased (*p* < 0.01) (4) The expression of Fas/FasL was decreased (*p* < 0.01)	[58]

Vascular dementia (VaD)	0.3 mL of 3% clot suspension was injected into the internal carotid artery, rats	Non-MA	CV17, CV12, CV6, T36, SP10, MA	(1) The TUNEL-positive cells decreased (*p* < 0.01) (2) Both the mRNA and protein expression of Bax were decreased (*p* < 0.01) (3) Both the mRNA and protein expression of Bcl-2 were increased (*p* < 0.01)	[59]

EA: electroacupuncture; MA: manual acupuncture; I/R: ischemia-reperfusion; TUNEL: terminal deoxynucleotidyl transferase-mediated dUTP-biotin nick end labeling; TNF-*α*: tumor necrosis factor-*α*; IL-1*β*: interleukin-1*β*; MMP-9: matrix metalloproteinase-9; LC3: microtubule-associated protein light chain; NDRG2: human N-Myc downstream-regulated gene 2; COX-2: cyclooxygenase-2; NK-1R: neurokinin 1 receptor; BrdU: 5-bromo-2′-deoxyuridine; ERK1/2: Ras-dependent extracellular signal-regulated kinase 1/2; CUS: chronic unpredictable stress; *ε*PKC: epsilon protein kinase C; TIMPs: tissue inhibitors of metalloproteinases; mTORC1: mammalian target of rapamycin complex 1; NF-*κ*B: nuclear factor-kappa B; TLR-4: toll-like receptor-4.
